# Amyloid Precursor Protein Changes Arrangement in a Membrane and Its Structure Depending on the Cholesterol Content

**DOI:** 10.3390/membranes13080706

**Published:** 2023-07-28

**Authors:** Vladimir D. Krasnobaev, Yaroslav V. Bershatsky, Olga V. Bocharova, Eduard V. Bocharov, Oleg V. Batishchev

**Affiliations:** 1 Frumkin Institute of Physical Chemistry and Electrochemistry, Russian Academy of Sciences, Leninsky Prospekt, 31, 119071 Moscow, Russia; vladimir-krasniy@yandex.ru; 2 Research Center for Molecular Mechanisms of Aging and Age-related Diseases, Moscow Institute of Physics and Technology, Institutski per., 9, 141701 Dolgoprudny, Moscow Region, Russia; bershackyjaroslav@gmail.com (Y.V.B.); edvbon@mail.ru (E.V.B.); 3 Shemyakin-Ovchinnikov Institute of Bioorganic Chemistry, Russian Academy of Sciences, Miklukho-Maklaya, 16/10, 117997 Moscow, Russia; o.bocharova@gmail.com

**Keywords:** Alzheimer’s disease (AD), amyloid precursor protein (APP), atomic force microscopy (AFM), amyloidogenic mutant L723P, amyloid beta (Aβ) peptides, lipid raft, lipid domain, cholesterol, protein-lipid interactions, membrane pores

## Abstract

One of the hallmarks of Alzheimer’s disease (AD) is the accumulation of amyloid beta (Aβ) peptides in the brain. The processing of amyloid precursor protein (APP) into Aβ is dependent on the location of APP in the membrane, membrane lipid composition and, possibly, presence of lipid rafts. In this study, we used atomic force microscopy (AFM) to investigate the interaction between transmembrane fragment APP_672–726_ (corresponding to Aβ_1–55_) and its amyloidogenic mutant L723P with membranes combining liquid-ordered and liquid-disordered lipid phases. Our results demonstrated that most of the APP_672–726_ is located either in the liquid-disordered phase or at the boundary between ordered and disordered phases, and hardly ever in rafts. We did not notice any major changes in the domain structure induced by APP_672–726_. In membranes without cholesterol APP_672–726_, and especially its amyloidogenic mutant L723P formed annular structures and clusters rising above the membrane. Presence of cholesterol led to the appearance of concave membrane regions up to 2 nm in depth that were deeper for wild type APP_672–726_. Thus, membrane cholesterol regulates changes in membrane structure and permeability induced by APP that might be connected with further formation of membrane pores.

## 1. Introduction

The most common cause of elderly related dementia is Alzheimer’s disease (AD) [1,2]. Although the precise etiology of AD is still not completely clear, it has been reported that it has a strong relation to the processing of amyloid precursor protein (APP)—a 770 amino acid protein expressed by neurons—and generating amyloid β (Aβ) peptides from it by the proteolytic cleavage [3]. One of the mechanisms of Aβ leading to forming senile plaques, correlating with AD in mice, was thoroughly investigated in [4]. The authors studied faulty autophagy, leading to the profusion of Aβ-positive autophagic vacuoles, consecutively packing into large membrane blebs forming flower-like perikaryal rosettes and evolving into senile amyloid-plaques. As for the processing of APP before forming Aβ peptides, its transmembrane section can be cleaved in two major ways: by ɑ- or β-secretase near membrane surface followed by γ-secretase within a lipid bilayer in both cases. The β-secretase pathway, producing a C-terminal C99 fragment of APP, is associated with the Aβ peptide maturation. The cleaved Aβ sections perform as intrinsically disordered proteins (IDP [5]). When inserted in a bilayer, they can apparently switch between ɑ- and β-conformations forming different species of oligomers, including toxic, while in the extracellular aqueous medium, they fold into amyloid-fibril structures with a large number of β-sheets [6,7].

Different Aβ isoforms, usually consisting of 38–43 amino acids [8], normally occur in a healthy human brain regardless of the age, and are apparently essential for the brain’s function, participating in synaptic signal transduction, neuroplasticity, and inflammatory response [7]. Nevertheless, Aβ amyloid fibrils are a key component of so-called senile plaques, strongly associated with AD [9,10], although the mechanism of this connection is still questioned [7,11,12,13]. Mutations in the APP gene are linked to rare familial forms of Alzheimer’s disease [14]. According to [15], it includes, for instance, D678N (D7N in Aβ) [16], E693G (E22G in Ab) [17], and V717I or V717F [18,19]. Specifically, the L723P mutation of APP is found in the Australian early onset AD pedigree [20]. It is located in the C-terminus of the transmembrane domain of APP. The mutation is out of the range of the γ-secretase cleavage sites, approximately at the level of the intracellular phase separation boundary of the plasma membrane in the ε-site. According to [20], it increases Aβ42(43) production by 40–90%. Notably, this mutation does not change Aβ42/40 ratio as much as other common mutations [21], but it completely blocks the production of amyloid intracellular domain (AICD) 50–99, which is normally produced in equal amounts with AICD 49–99 for wild type of APP. The change of the transmembrane domain position in the membrane corresponding to L723P mutation is, thus, considered to alter the activity of the γ-secretase complex. The distinct mechanism for L723P is specifically described in [22]: L723P mutation causes local unfolding of the C-terminal turn of APP transmembrane domain helix and increases its accessibility to water required for cleavage of the protein backbone by γ-secretase in the ε-site; thus, the sequential proteolysis of C99 is enhanced, resulting in accumulation of the pathogenic forms of amyloid-β.

Notably, APP processing in this case differs depending on its localization in the raft or non-raft part of the membrane: ɑ-secretase cleaves APP located in the non-raft phase of the membrane, and β-secretase BACE-1 and γ-secretase cleaves the APP in the raft phase [23]. These parts of a membrane, especially in the context of model membranes, are often referred to as liquid-ordered (L_o_) and liquid-disordered (L_d_) phases for the raft and non-raft parts, respectively. More than a half of the mutations of the APP family predisposing to AD occur precisely in its transmembrane domain, approximately corresponding either to the residues 700–723 of APP (e.g., V717I/F, A713V, T714I/A, V717F/I/L/G, and L723P in particular), or to the juxtamembrane region 688–694, which is located right after the metal-binding domain in the form of a flexible alpha-helix (e.g., mutations A692G, E693Q/K/G, D694N) [7,19,21,24].

It is known that Aβ chains have the property of binding to glycosphingolipids and cholesterol [25,26]. As for the APP, its fragment APP_672–770_, often referred to as C99, was also studied to be a cholesterol-binding site [27], especially the loop region connecting its juxtamembrane helix and N-terminal part of transmembrane helix [26,28,29]. It was later shown that indeed different mutations in the transmembrane domain of APP significantly affect cholesterol binding [30].

We previously studied lipid rafts as formations with a certain boundary area and line tension (energy of the domain boundary per unit length of its perimeter) in a membrane [31]. We also investigated how molecules of various molecular geometry influence raft formation [32,33], acting as so-called line active components [34] and made an analysis of interplay between lipid domains and aging-related diseases [35]. Here, we used atomic force microscopy to investigate the interaction between the fragment APP_672–726_ (corresponding to Aβ_1–55_) and model membranes containing L_o_ lipid domains with different concentrations of cholesterol for wild-type (WT) and L723P-mutant forms of APP. We believe that studying APP in an environment close to the lipid matrix of cell membrane can improve our understanding of APP binding to cholesterol and lipid rafts.

## 2. Materials and Methods

The lipids for the experiments, namely 1,2-Dioleoyl-sn-glycero-3-phosphocholine (DOPC), egg sphingomyelin (eSM), and cholesterol (Chol), were bought from Avanti Polar Lipids (Alabaster, AL, USA). The solvents for the lipids, namely methanol (>99.0%) and chloroform (>99.0%), were purchased from Sigma-Aldrich (St. Louis, MO, USA). WT and L723P (mutant) APP_672–726_ fragments were produced using cell-free expression as described in [36]. The chemicals were used without additional purification. For following liposome preparation, DOPC and Chol in powder form were dissolved in chloroform to the final concentration of 10 g/L, eSM was dissolved in chloroform/methanol (9:1, *v*:*v*) to the concentration of 5 g/L.

To prepare the lipid films with APP_672–726_, the components were combined in a given molar ratio. The mixture was then placed in a glass vial and subjected to rotary evaporation under low vacuum conditions (400 mbar) at a temperature of 36 °C for a duration of 10 min. Afterward, it remained under high vacuum conditions (40 mbar) for an additional 30 min to ensure thorough drying. The resulting dried lipid film was reconstituted in deionized water (with a resistivity of 18.2 MΩ·cm), resulting in a final lipid concentration of 0.5 g/L. To facilitate the formation of small vesicles, the vial containing the lipid suspension was subjected to sonication for 30 min while maintaining a temperature of 50 °C. The vesicle suspensions were utilized immediately after preparation.

The AFM experiments were conducted using the Multimode Nanoscope V (Bruker, Billerica, MA, USA) setup, which was equipped with an electrochemical fluid cell. Initially, a 100 μL sample of the heated vesicle suspension was placed onto a freshly cleaved mica surface and allowed to incubate for 5 min. During this time, the vesicles ruptured and formed a lipid film on the mica surface. Subsequently, the lipid film was rinsed thoroughly with room temperature Milli-Q (Merck, Darmstadt, Germany) water, repeating the process three times to eliminate any lipid multilayers. The rinsed lipid film was then transferred to a fluid cell for further AFM imaging. To facilitate imaging, 50 μL of Milli-Q water was added to the cell. Silicon-tip on Nitride Lever (SNL-10) cantilevers with a nominal elastic constant of 0.06 N/m and a tip radius of approximately 2 nm (Bruker, Billerica, MA, USA) were employed for the experiments. The images were scanned at dimensions of 3 × 3 μm^2^ and 10 × 10 μm^2^ and subsequently processed using WSxM software [37].

To analyze the size of individual liquid-ordered domains and, thus, detect a change in the line tension, we employed a method outlined in [32]. In summary, as the shape of domains was not perfectly circular and exhibited merging over time, the area of individual domains could vary. The boundaries of these domains were a combination of circular arcs, reflecting their initial sizes and could be approximated by corresponding circles. Consequently, we overlapped circles of different diameters onto the liquid-ordered phase domains near their boundaries to determine the best match.

## 3. Results

### 3.1. APP and Lipid Rafts

We conducted a series of experiments for APP_672–726_ fragments in supported lipid membranes with the physiologically relevant 1 mol. % of APP_672–726_, the fixed molar ratio of DOPC/eSM = 1/1 and a variable amount of cholesterol (10%, 20%, and 33%, in mol.). We made the same experiments for the WT and for the mutant. Each bilayer was scanned for at least 2 h to detect any changes in time. We also conducted experiments with 40 mol. % Chol content, but we did not obtain persistent results, because we were not able to obtain stable supported bilayers with the same protocol. We tried to determine whether APP can act as a line active component like lysolipids and GM1 [32,33], and how it correlates with the amount of cholesterol in a membrane.

**Figure 1:**
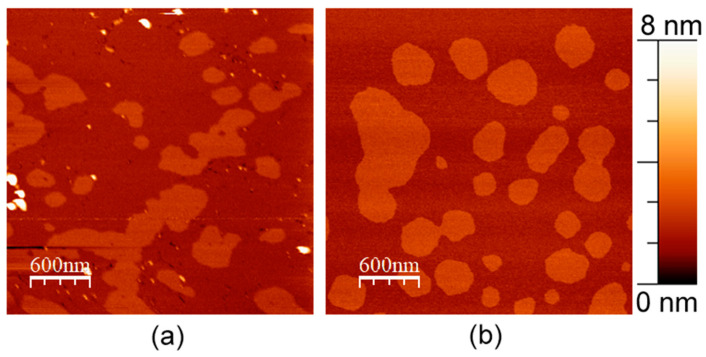
DOPC/eSM = 1:1 with 20 mol. % Chol supported lipid bilayer. (**a**) With 1 mol. % of APP L723P; (**b**) without APP. Size of each image is 3 μm × 3 μm.

In general, we observed single immobilized fragments of APP slightly rising above the membrane for several nanometers, as well as their clusters (Figure 1a). Precisely, in most cases, we observed different immobilized clusters and concavities in the membrane. APP clusters had a relatively wide range of heights and shapes, but it is accurate to say that they had a characteristic height of about 1.5 ± 0.4 nm above the membrane and a diameter of about 20 ± 10 nm. Concavities had an average diameter of 30 ± 10 nm and were up to 2 nm in depth appearing only in presence of APP in the membrane (Figure 1). For comparison, the topographies of such lipid bilayers without APP usually appear as flat surfaces with minor impurities, such as pieces of multilayers or gaps in the supported layer, which we also observed in these experiments (Figure 1b). These impurities without APP are very mobile, as they are mostly removed eventually by moving cantilever along the membrane, due to its lateral fluidity.

**Figure 2:**
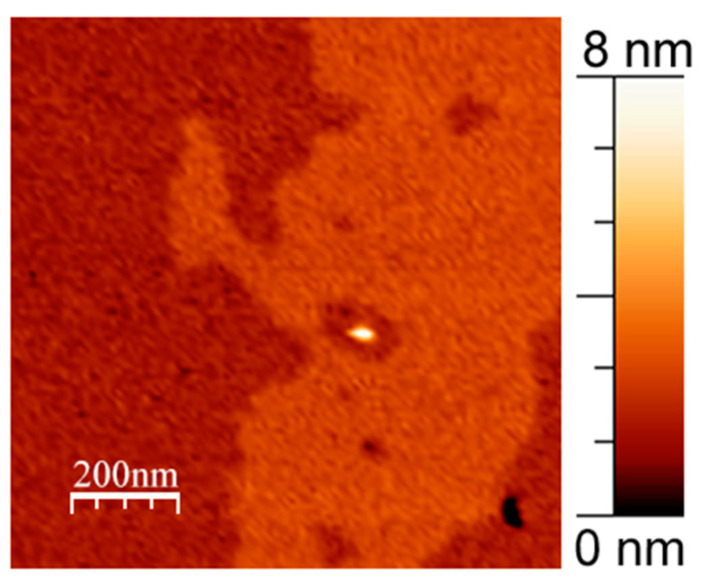
APP cluster within the raft surrounded by a small region of L_d_ phase. 1 mol. % WT in DOPC/eSM = 1:1 with 33 mol. % Chol supported lipid bilayer. Size of the image is 1 μm × 1 μm.

Despite the domain pattern potentially looking different in the presence of APP, we did not detect any major changes in the curvature of the domain boundary for any membrane composition. It is known that domains might merge over time; therefore, we considered the curvature of their boundary being a more important and objective parameter of the typical domain size [32,33]. We did not detect APP clusters or single fragments inside L_o_ lipid domains in any experiment for both WT APP and its L723P mutant. Even when we observed clusters within the L_o_ phase, they were surrounded by a small region of the L_d_ phase inside the raft (Figure 2).

### 3.2. APP and Cholesterol Content

**Figure 3:**
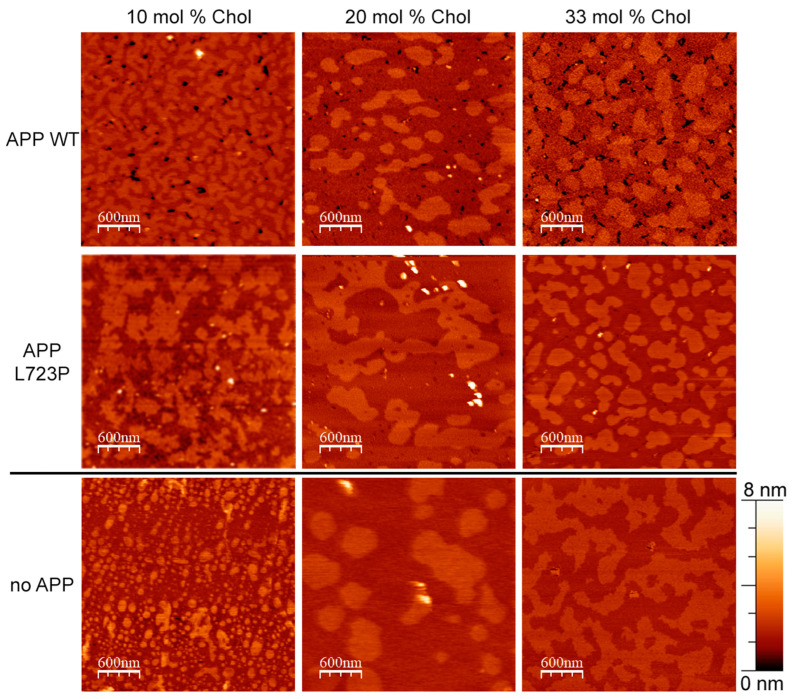
AFM images of supported lipid bilayers with different lipid compositions with and without APP. Type of the APP added is indicated for each row. The amount of cholesterol in the DOPC/eSM = 1:1 membrane is indicated for each column. The size of images is 3 μm × 3 μm.

As the interaction of different APP fragments with cholesterol also seems to be an important factor in its processing [7,27,28,30,38,39,40], we decided to investigate if it would be possible to detect any difference in APP clusterization and APP-induced changes in a membrane structure for different concentrations of cholesterol in the lipid bilayer. The set of corresponding topographies is displayed in Figure 3.

We assessed the number of APP clusters and APP-induced concave membrane regions, recognizable as single, per area. For each composition, we also measured the average size of the APP cluster. The results are displayed in Table 1. Without added APP, the concavities were not detected. Through holes in the bilayer were present occasionally even without APP, but they were usually resealed after 30 min of scanning. In the presence of APP, through pores were stable for at least 2 h of scanning.

Here, we should note that the method of measurement was restricted at least by the sizes of the cantilever tip (2 nm diameter in our case), and this made it impossible to determine structures of smaller sizes to be a single APP fragment or have any inner structure. Still, the parameter of “clusters, recognized as separate” is arguably useful for comparison within the experiments.

As seen in Table 1, the formation of clusters and concave membrane regions for WT APP did not depend on the amount of cholesterol. In contrast, for the mutant APP, we clearly observed a gradual decrease in the number of separate clusters, their average height and an increase in the number of concave membrane regions correlating with the increase in the mole fraction of cholesterol. Depth of the concave regions was two times greater for WT APP comparing to its L723P mutant.

### 3.3. APP in a Membrane without Cholesterol

**Figure 4:**
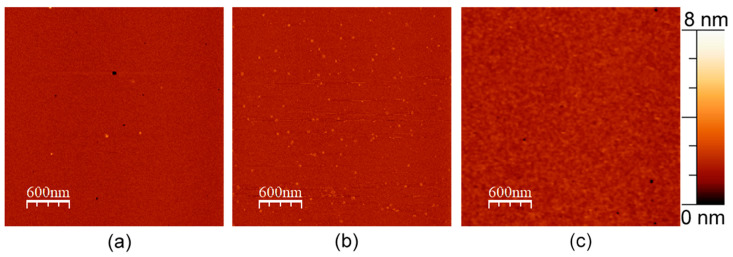
AFM images of DOPC bilayers with (**a**) 1 mol. % of WT APP, (**b**) 1 mol. % of L723P APP, and (**c**) no APP added. The size of images is 3 μm × 3 μm.

We also performed experiments with 1 mol. % APP in a pure DOPC bilayer (Figure 4). We did not perform experiments with APP in DOPC/eSM = 1:1 bilayer without cholesterol, as at such conditions, the bilayer separates into DOPC bilayer and eSM gel domains, which are out of our interest for this study.

**Figure 5:**
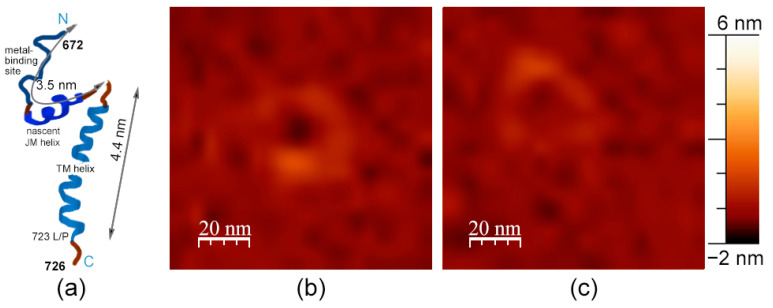
(**a**) Structure of the APP molecule. (**b**) Rings of APP in DOPC bilayer with 1 mol. % of WT APP. (**c**) Rings of APP in DOPC bilayer with 1 mol. % of L723P mutant. The size of the images (**b**) and (**c**) is 100 nm × 100 nm.

For both WT and L723P APP, we observed APP fragments and small fraction of annular clusters, which we did not observe in the presence of even 10 mol. % of cholesterol in a membrane (Figure 5b,c). All these rings had the diameter of 30 ± 5 nm and the height of 1.5 ± 0.5 nm for both WT and L723P APP. The bilayer with WT APP also had small pore-like holes with the same average diameter, sometimes located within an amyloid ring. As for L723P rings, there always was a clear bilayer within them. For both experiments, we can see that the rings consisted of subunits, and each ring was about 90 ± 15 nm of circumference. Taking into account that the length of the juxtamembrane and metal-binding part of the APP_672–726_ together was about 3.5 nm (Figure 5a) [41], we expected about 25 ± 5 molecules in the ring.

## 4. Discussion

As described previously, amyloid processing depends on its location in a liquid-ordered or liquid-disordered phase of a membrane [8]. However, we did not notice any major changes in the raft structure that would be induced by APP in a series of phase-separating membranes with different cholesterol content. On the other hand, in every conducted experiment, we observed the disproportional amount of APP fragments being located either in the liquid-disordered phase or at the boundary between ordered and disordered phases, and hardly ever in the domains. We emphasize that a slightly larger domain of APP, precisely C99 domain (APP_672–770_), was previously proven to perform as a cholesterol binder [27]. Thus, our study provides an extra piece of evidence that APP interaction with cholesterol in a membrane may be a more impactful factor for APP processing than the interaction with lipid rafts, as suggested in [27,39]. In addition to this proposition, we also know that the mutation of raft targeting in BACE1 did not influence APP processing in cell culture studies and BACE1 could cleave APP in both raft and non-raft domains [42]. Similarly, identified raft targeting signals in γ-secretase complex subunits, nicastrin, and APH1 had no impact on the association of the mature γ-secretase complex with rafts, nor did it affect APP processing to Aβ [43].

In membranes without cholesterol, we noticed that APP molecules aggregate in annular structures. We did not detect these structures in any other membrane composition. Notably, annular structures formed by Aβ peptides are widely shown in literature [44,45,46,47]. Nevertheless, we did not find any studies discovering or investigating annular structures, formed with parts of APP having helical transmembrane domain, as we did in this study. In the case of WT APP, these rings had a hole inside them that might increase membrane permeability because of the decrease in a membrane thickness. Interestingly, for amyloidogenic L723P, we did not observe such holes; therefore, it hardly influenced the membrane structure in absence of cholesterol. Moreover, we can recognize around six subunits in the APP rings (Figure 5b,c). As we assessed the number of molecules to be around 25, the total size of the APP ring suggests that these subunits are not single molecules but molecule clusters. We can propose other organization schemes for the APP in the rings. For instance, if the juxtamembrane helix folds into a β-strand, the free length of the outer part is about 2 nm, which would fit 45 ± 8 molecules in a ring. If the transmembrane helix (4.4 nm) is on the membrane surface, that would fit 11 ± 2 molecules in a ring.

In every experiment for the same lipid composition, the presence of the L723P mutant of APP was more noticeable quantitatively than the presence of WT APP. The average number of observed separate “hills” per unit area corresponding to the APP clusters was recognizably higher for L723P than for WT in the same membrane composition (Table 1). We think that it reflects the fact that L723P is an amyloidogenic mutation, making an APP molecule more accessible to secretase cleavage, thus rising above the lipid bilayer higher than for WT APP. As for concave regions, we registered a higher number of separate concavities per unit area for WT compared to L723P mutant up to the cholesterol content of 33 mol. % cholesterol, where they were almost the same. This correlates with the fact that WT APP formed holes in a membrane even in the absence of cholesterol, and suggests that the increase in the cholesterol content in a membrane led to the appearance of APP-induced membrane holes, increasing membrane permeability. We should emphasize that these holes are not membrane pores, because their depth did not exceed 2 nm. However, their presence in a membrane unambiguously makes it more prone to spontaneous pore formation.

**Figure 6:**
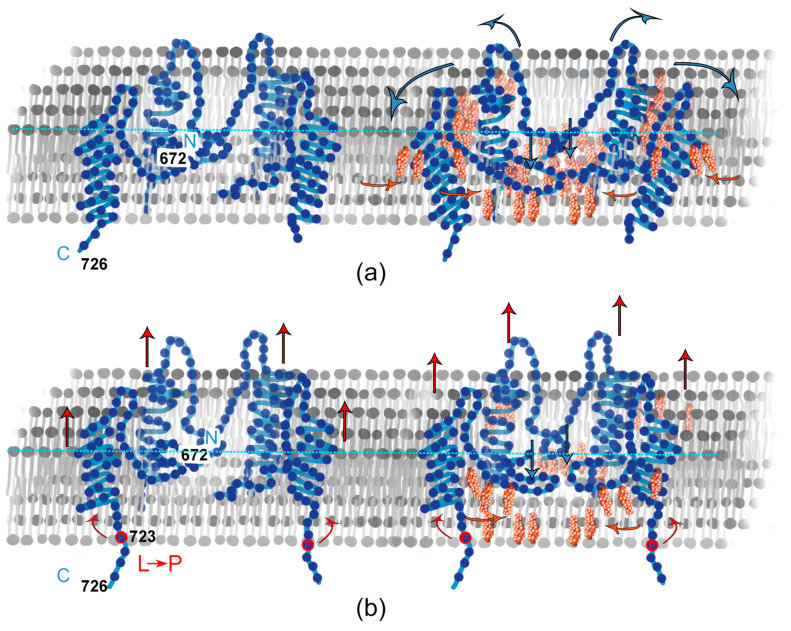
Scheme of interaction of APP and Chol in a bilayer. (**a**) WT; (**b**) L723P.

We noticed that WT APP formed relatively deep holes, whose number per unit area was almost independent of the concentration of cholesterol, unlike L723P APP, for which we observed less clusters and more concavities with an increase in the cholesterol concentration. We suggest that the physicochemical mechanism of this APP behavior is as follows. The WT APP forms clusters and concavities with N-domain, binding cholesterol if available, while the amyloidogenic L723P mutation places the molecule a little bit higher above the membrane, while binding of cholesterol still makes it immerse deeper into the membrane but to a lesser extent. Moreover, for WT APP, the presence of cholesterol induces concave regions as deep as one lipid monolayer, and such strong membrane re-structuring requires increase in the lateral diameter of the APP-induced membrane hole (Figure 6).

We also assumed that, in our experiments with supported lipid bilayers, the structure of a transmembrane domain of APP prevents ordering of lipids, making APP to be present exclusively in the L_d_ phase and explaining the structural features presented in Figure 2. Notably, the cholesterol-binding sites of the APP are known to be located from 697 to 696 and from 700 to 710 [27,29,48], the mutations investigated in the literature that affect cholesterol binding were from 691 to 710 [28], while the mutation investigated by us was in position of 723. We suspect that amyloidogenic L723P mutation may change the positioning of the transmembrane domain of APP in the membrane [22], consecutively affecting the positioning of the cholesterol-binding site, as it is normally placed in the extracellular and the juxtamembrane part of the transmembrane domain. That means that apart from the specifical cholesterol binding site in the domain, which is studied in the literature [27,39], its interaction with cholesterol is also indirectly impacted by the mutation investigated here. Also, we noticed that the WT APP formed relatively stable concavities in the bilayers that was not common for neither the membrane without APP, nor for L723P mutant of APP. In general, we observed that the detectable presence of L723P domains decreased with cholesterol, and at the same time, the number of holes in a membrane increased. So, firstly, we observed that the more cholesterol is available for L723P APP, the deeper in the membrane it acts, creating more craters, while WT APP membrane activity is independent of the cholesterol content. Interestingly, studies showed that the dependance of amyloid production on cholesterol content is complex because cholesterol apparently does not only influence APP organization in a membrane, but also influence the activity of BACE1 [49]. Thus, our study contributes to understanding the effect of cholesterol on APP in a membrane, excluding the influence of BACE1. Secondly, with no cholesterol in the membrane, APP_672–726_ forms annular structures, very similar to annular structures formed by Aβ peptides. Therefore, we might suggest that immersing of the APP fragments in a membrane prevents formation of amyloid Aβ peptides, and cholesterol performs a protective role. However, it simultaneously increases membrane permeability, making them more vulnerable for pore formation.

## Figures and Tables

**Table 1:** The average quantity of APP-induced formations and the heights of APP clusters specifically in DOPC/eSM = 1:1 membrane with differing cholesterol content. Each value represents an average and a standard deviation, derived from measurements of at least 20 clusters or concavities obtained from each sample.

APP Type	Type of Structure	Parameter	10 mol. % Chol	20 mol. % Chol	33 mol. % Chol
WT	Clusters	quantity, units/μm^2^	2 ± 1	4 ± 1	2 ± 1
		average height, nm	3 ± 2	4 ± 2	4 ± 2
	Concavities	quantity, units/μm^2^	12 ± 3	10 ± 3	14 ± 3
		average depth, nm	1.8 ± 0.4	2.0 ± 0.5	2.8 ± 0.5
L723P	Clusters	quantity, units/μm^2^	15 ± 3	6 ± 2	7 ± 2
		average height, nm	4 ± 2	3 ± 2	3 ± 2
	Concavities	quantity, units/μm^2^	4 ± 1	8 ± 1	15 ± 3
		average depth, nm	0.8 ± 0.2	0.8 ± 0.2	0.9 ± 0.2

## Data Availability

Data will be made available upon reasonable request.
